# Advertising Dentists

**Published:** 1900-06-16

**Authors:** 


Vol. XXVIII.—No. 716.] Edited by Sir Henry Burdett, K.C.B., and Solomon C. Smith, M.D., M.R.C.P. [June 16, 1900.

### Advertising Dentists.

A letter appeared in the *Times* of the 9th instant, signed by a gentleman who cddly enough described himself as "the *honourable* general secretary of the London and Counties Medical Protection Society," in which it was set forth that the General Medical Council, during its recent session, had decided "to the effect" that an attempt (presumably by a dentist) to "attract practice by means of advertisements" is, in the opinion of the Council, "infamous conduct in a professional respect." It was also set forth that, at the same meeting, the name of a dentist was directed to be erased from the Dental Register "for the above offence." Considering that Mr. Justice Henn Collins, in his well-known decision in the case of Allinson, laid down that it could not be considered "infamous conduct," even in a medical practitioner, to advertise his calling, unless the advertisements themselves were of an improper or disgraceful character, the announcement in question was somewhat startling, and excited in our minds a spirit of inquiry which led to an examination of the minutes of the Council meeting. From these it appears that a Mr. Owen McBreen, who had been registered in the Dentists' Register as having been in practice before July 22nd, 1878, but who possessed no other registerable qualification, not being a licentiate or member of the Royal College of Surgeons of England, Ireland, or Edinburgh, or of any such colleges, was found by the Dental Committee of the Council, on formal inquiry, "habitually to have published advertisements in which he described himself as R.D.S.E. (registered by the Royal College of Surgeons), thereby causing it to be inferred that he is connected with one of the Royal Colleges." On this report, it being the statutory business of the Committee in question to "find the facts" for the information of the Medical Council, the Council deliberated, and, after a very patient hearing of Mr. McBreen in his defence, they resolved that "in the facts found in the report of the Dental Committee it has been proved that Owen McBreen has been guilty of conduct which is infamous or disgraceful conduct in a professional respect." Upon this the Registrar was directed to erase the name of Mr. McBreen from the Dentist's Register. In another case, which would appear to be the one referred to in the letter, one Arthur Oglesby, also registered without qualification as a dentist who had been in practice before 1878, was found by the Committee to have used in his advertisements the letters "D.D.S., Un. Ill.," and it was also found that the advertisements were in other respects of a "highly objectionable" character. The letters were supposed to refer to an American diploma which Oglesby admitted to have been obtained by purchase, without visiting America, or being subjected to any examination; and it was shown that in 1878, when he represented himself as being in actual practice as a dentist, he was only sixteen years of age. In neither case, it will be observed, was the offence merely what was described by the "honourable" secretary, that is to say, an attempt to attract practice by means of advertisements, but it was an attempt to assume a character to which the advertiser was not entitled.

The truth is that the question of advertising by dentists has been carefully considered by the Medical Council, in connection with the statutory limitations of its powers. There can be no doubt that the practice is exceedingly objectionable in itself, or that it has been carried to most offensive lengths. There are dentists in some of the northern manufacturing towns whose houses are covered over with gigantic posters, bearing fancy portraits of sufferers from toothache, sketches of sets of artificial teeth, or of jaws collapsed from the absence of these useful appendages, and rejuvenescence on the part of those to whom they have been supplied. It is, of course, open to the Royal Colleges to grant their dental diplomas or licences on their own conditions, and to stipulate that the holders of them shall refrain from advertising, on pain of forfeiture, which would entail removal from the Register. But, with regard to persons who were registered without qualifications, as having been in practice before 1878, the case is entirely altered. The Medical Council cannot restrain them from advertising, unless the advertisements themselves are of an objectionable character. On this ground, the Council some years ago cautioned dentists that the practice of advertising, besides being highly derogatory to persons who assumed to be members of a learned profession, might easily be carried to such a length, by the assertion of absurd claims of superiority to other people, or by equally absurd depreciation of them, as to amount to infamous conduct. In other words, the Council pointed out that it might be infamous to seek to attract patients by lying; and this view they have acted upon. The courts of law would never permit a highly penal statute to be interpreted in any but the most rigid manner, and there is not the slightest probability that the simple act of advertising by dentists will ever be pronounced by the Council to be infamous or disgraceful in a professional respect. Fortunately, twenty-two years have already elapsed since the Dentists' Act came into operation, and the practitioners prior to 1878 must now be a small and rapidly-vanishing minority. Their complete disappearance will open the way for a new generation, whose conduct in this respect will be under the control of the corporations from which their qualifications are derived.

